# Efficacy and safety of semaglutide injection in Indian patients with type 2 diabetes mellitus inadequately controlled on metformin: a phase 3, randomized, active-controlled trial (SIZE-DM study)

**DOI:** 10.1186/s40842-026-00290-8

**Published:** 2026-04-24

**Authors:** Nitin Kapoor, Shehla Shaikh, Saptarshi Bhattacharya, Sanjay Kalra, Sambit Das, Sunil Kota, Vipul Khandelwal, Arindam Naskar, Amol Dange, Sanket Sorate, Mayura Choudhari, Narayan Deogaonkar, Jayashree Shembalkar, Deepak Varade, Shejole Vivek Samadhan, Shrikant Somani, Richa Giri, Jasminder Singh, Rajesh Kumar Palaparthi, Narendra Prasad, Paramesh Shamanna, Santosh Saklecha, Kushal Bangar, Aruna Mangipudi, Mayur Mayabhate, Nitin Kapure, Akhilesh Sharma, Radhakrishna Vaddem, Mukesh Jaiswal

**Affiliations:** 1 Christian Medical College (CMC), Vellore, Tamil Nadu India; 2 Prince Aly Khan Hospital, Mumbai, Maharashtra India; 3 Indraprastha Apollo Hospitals, New Delhi, India; 4 Bharti Hospital, Karnal, Haryana India; 5 Kalinga Institute of Medical Sciences, Bhubaneswar, Odisha India; 6 Diabetes & Endocrine Clinic, Berhampur, Odisha India; 7 Apex Hospital Pvt. Ltd., Jaipur, Rajasthan India; 8 School of Tropical Medicine, Kolkata, West Bengal India; 9 Lifepoint Multispecialty Hospital, Pune, Maharashtra India; 10 Sanjeevani Criticare and Research Centre Pvt. Ltd., Nashik, Maharashtra India; 11 Ishwar Institute of Health Care, Chhatrapati Sambhajinagar, Maharashtra India; 12 Deogaonkar Multispeciality Hospital, Nashik, Maharashtra India; 13 Getwell Hospital and Research Institute, Nagpur, Maharashtra India; 14 Shree Ashirwad Hospital, Dombivli, Maharashtra India; 15 Medipoint Hospitals Pvt. Ltd., Pune, Maharashtra India; 16 Navneet Memorial Hospital “Sushrusha”, Ahmedabad, Gujarat India; 17 G.S.V.M. Medical College, Kanpur, Uttar Pradesh India; 18 Graduate Institute of Medical Sciences (PGIMS), Rohtak, Haryana India; 19 Government Siddhartha Medical College, Vijayawada, Andhra Pradesh India; 20 Sparsh Superspeciality Hospital, Bangalore, Karnataka India; 21 Bangalore Diabetic Centre, Bangalore, Karnataka India; 22 Santosh Hospital, Bangalore, Karnataka India; 23 AIMS Hospital, Dombivli, Maharashtra India; 24 King George Hospital, Visakhapatnam, Andhra Pradesh India; 25 Medical Affairs and Clinical Research Division, Alkem Laboratories Ltd., Maharashtra Mumbai, 400063 India

**Keywords:** Semaglutide, Type 2 diabetes mellitus, GLP-1 receptor agonist, Non-inferiority, Indian population, HbA1c reduction

## Abstract

**Background:**

This study evaluated the efficacy and safety of generic semaglutide compared with innovator Semaglutide in Indian adults with type 2 diabetes mellitus (T2DM).

**Methods:**

This Phase 3, multicenter, randomized, active-controlled, non-inferiority trial enrolled 320 adults with T2DM inadequately controlled on metformin. Participants were randomized 1:1 to receive either generic semaglutide (Alkem laboratories Ltd.) or innovator Inj. semaglutide (Novo Nordisk) for 24 weeks in step-wise dose escalation from 0.25 mg/week to 2 mg/week. The primary endpoint was change in HbA1c from baseline to Week 24. Secondary endpoints included changes in fasting and post-prandial glucose, body weight, and proportion of patients achieving HbA1c < 7.0%. Safety assessments included adverse events, hypoglycemia, various laboratory parameters.

**Results:**

Of 320 participants randomized, 313 completed the study. Baseline demographic and clinical characteristics were comparable between groups. At Week 24, both treatments achieved significant HbA1c reductions (mean − 2.20%), with generic semaglutide demonstrating non-inferiority to the reference. Reductions in body weight, fasting and post-prandial glucose were similar between arms. A total of 86.62% of participants achieved HbA1c < 7.0%. Safety profiles were comparable, with predominantly mild-to-moderate adverse events and no treatment-related serious adverse events.

**Conclusion:**

Generic semaglutide demonstrated non-inferior efficacy and comparable safety to innovator Semaglutide in Indian adults with T2DM inadequately controlled on metformin, offering an effective and accessible therapeutic option in resource-limited settings.

**Supplementary Information:**

The online version contains supplementary material available at 10.1186/s40842-026-00290-8.

## Introduction

Type 2 diabetes mellitus (T2DM) is a progressive metabolic disorder characterized by insulin resistance and β-cell dysfunction, contributing to chronic hyperglycemia and increased risk of cardiovascular and renal complications. India bears a disproportionate burden of T2DM, with over 100 million affected individuals, and faces unique challenges in diabetes care due to socioeconomic disparities, limited access to advanced therapies, and high out-of-pocket expenditures [1].

Glucagon-like peptide-1 receptor agonists (GLP-1RAs) have emerged as a cornerstone in modern diabetes management, offering glycemic control, weight reduction, and cardiovascular protection. Semaglutide, a long-acting GLP-1RA administered once weekly, has demonstrated superior efficacy across the SUSTAIN and STEP trial programs. These trials consistently showed significant reductions in HbA1c, body weight, and major adverse cardiovascular events (MACE) in patients with T2DM and obesity [2–4].

The SELECT trial, a landmark cardiovascular outcomes study, extended semaglutide’s benefits to individuals with obesity and established cardiovascular disease, even in the absence of diabetes. It reported a 20% reduction in MACE and sustained weight loss over four years, with favorable renal outcomes and reduced albuminuria [2, 5]. Meta-analyses further confirmed semaglutide’s impact on reducing cardiovascular mortality, heart failure hospitalizations, and non-fatal myocardial infarctions [6].

Despite these global advances, data on semaglutide use in Indian populations remain limited. The high cost of the innovator semaglutide poses a significant barrier to accessibility, especially in low- and middle-income settings where affordability directly influences treatment adherence and outcomes [1]. Local manufacturing of generic formulations may offer a viable solution to improve access without compromising efficacy or safety.

This Phase 3, multicenter, randomized, single-blind, active-controlled, non-inferiority trial was designed to evaluate the efficacy, safety, and immunogenicity of generic semaglutide formulation compared to innovator semaglutide in Indian adults with T2DM inadequately controlled on metformin. The study aimed to generate robust local evidence to support the clinical utility and affordability of semaglutide in India.

## Methods

This Phase 3, multicenter, randomized, active-controlled, parallel-group, non-inferiority trial conducted across 23 tertiary care centers in India to evaluate the efficacy and safety of generic Inj. semaglutide (Alkem laboratories Ltd) compared to innovator Inj. semaglutide (Novo Nordisk) in patients with T2DM. The study adhered to the principles outlined in the Declaration of Helsinki, ICH-GCP E6(R2), and applicable Indian regulatory guidelines. Ethics approval was obtained from institutional review boards at all participating sites, and written informed consent was obtained from all participants. The trial is registered with the Clinical Trials Registry of India (CTRI/2025/02/081490) on 28/02/2025.

Eligible participants are adults aged 18–65 years, diagnosed with T2DM for at least six months, with HbA1c levels between 7.0% and 10.0%, and inadequate glycemic control despite receiving ≥ 1000 mg/day of metformin for at least 8 weeks were enrolled. Key exclusion criteria included type 1 diabetes, diabetic ketoacidosis, eGFR < 45 mL/min/1.73 m², significant hepatic impairment, history of pancreatitis or multiple endocrine neoplasia type 2 (MEN2), diabetic retinopathy, thyroid dysfunction, recent cardiovascular events, serum calcitonin > 50 ng/L, and pregnancy or lactation.

Participants are randomized in a 1:1 ratio using Interactive Response Technology (IRT), stratified by baseline HbA1c levels. A single-blind design was employed, in which investigators were blinded to treatment allocation. At every investigational site, an unblinded study coordinator managed investigational product dispensing, provision of dosing instructions, administration training to participants, and maintained investigator blinding throughout the study period. Moreover, outcome assessors, including laboratory personnel measuring HbA1c, fasting plasma glucose, and body weight, as well as personnel grading adverse events, remained blinded to treatment allocation throughout the study.

Study drugs are administered subcutaneously once weekly with dose escalation as follows:

### Week 1–4

All patients to receive the initial dose of 0.25 mg once weekly for 4 Weeks.

### Week 4–8

All patients to be up-titrated to the dosage of 0.5 mg once weekly.

**At week 8**:

- Patients with HbA1C < 7.5%, continue to receive 0.5 mg dosage.
- Patients with HbA1C of ≥ 7.5%, to be up-titrated to a dose of 1 mg one weekly for next 4 weeks (week 8–12).

**At weeks 12**,** 16 and 20**:

- Patients with HbA1C < 7.5%, to continue to receive the ongoing dose.
- Patients with HbA1C of ≥ 7.5% and ongoing dose 0.5 mg, to be up-titrated to a dose of 1 mg.
- Patients with HbA1C of ≥ 7.5% and ongoing dose of 1 mg to be up-titrated to a dose of 2 mg.
- Patients with HbA1C of ≥ 7.5% and ongoing dose of 2 mg, to continue the same dose and to receive rescue medication as per the rescue medication criteria.

All patients continued metformin at their pre-study dose throughout the 24-week treatment period.

The primary efficacy endpoint was the change in HbA1c from baseline to week 24. Secondary endpoints included changes in fasting and post-prandial glucose levels, body weight, and the proportion of patients achieving HbA1c < 7.0% at week 24. Safety assessments included treatment-emergent adverse events (serious and non-serious), changes in clinical laboratory parameters, incidence of hypoglycemia requiring intervention, and development of anti-drug antibodies (ADA) and neutralizing antibodies from baseline to week 24.

Approximately 320 patients randomly assigned to either of the two treatment groups in this study, in a ratio of 1:1 (160 patients assigned to each of the 2 treatment arms). The sample size was calculated considering 15% dropout rate with 85% power to establish non-inferiority between the groups with one sided test at 95% CI, standard deviation (SD) from baseline changes of HbA1c as 1.1 with true treatment mean difference of 0 and non-inferiority margin as 0.4.

Efficacy analyses are conducted on the modified intent-to-treat (mITT) and per-protocol (PP) populations. Safety analyses include all patients who received at least one dose of study medication. The primary endpoint is analyzed using ANCOVA with baseline HbA1c as a covariate. Non-inferiority is concluded if the upper bound of the 95% confidence interval (CI) for the treatment difference is < 0.4%. Secondary continuous outcomes are analyzed using ANCOVA or mixed models, while categorical outcomes are assessed using chi-square or Fisher’s exact tests.

## Results

**Fig. 1:**
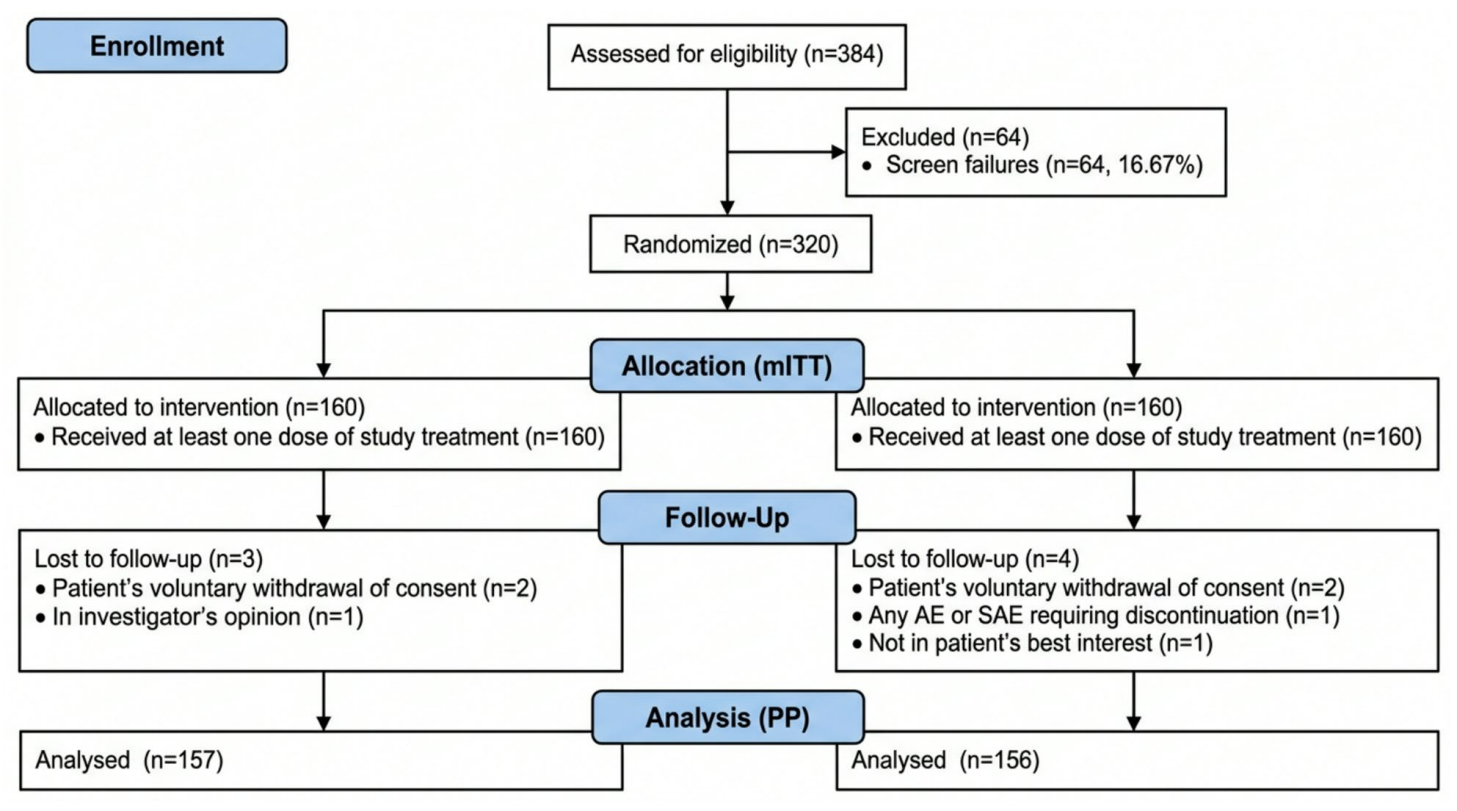
Consort diagram showing the flow of participants through the study

Of the 384 subjects screened, 320 were randomized in a 1:1 ratio to receive either generic semaglutide (test arm) or innovator semaglutide (reference arm). A total of 313 participants (98.13% in test arm; 97.50% in reference arm) completed the study per protocol. All randomized subjects (*n* = 320) were included in the safety population, with 160 subjects in each arm (Fig. 1).

The study population comprised 187 males (58.44%) and 133 females (41.56%). At baseline, the mean age was 50.07 ± 9.02 years, mean body weight was 70.93 ± 11.91 kg, and mean BMI was 27.42 ± 4.00 kg/m². Baseline demographic and clinical characteristics were comparable between the two treatment groups (Table 1).

**Table 1:** Demographic characteristics of study participants

Parameter	Test (*N* = 160)	Reference (*N* = 160)
Age (years)	49.66 (8.92)	50.97 (9.14)
Gender (M/F %)	59.38/40.62	57.50/42.50
Weight (Kgs)	71.11 (12.41)	70.75 (11.44)
Height (cm)	160.51 (8.21)	161.03 (7.96)
BMI (kg/m2)	27.54 (3.85)	27.30 (4.16)
Duration of Diabetes (Months)	51.56 (47.98)	49.83 (48.59)
HbA1C (%)	8.41 (0.77)^#^	8.47 (0.77)^#^
Fasting Plasma Glucose (mg/dL)^#^	162.61 (56.06)^#^	167.34 (68.24)^#^
Post-Prandial Plasma Glucose (mg/dL)^#^	226.80 (66.06)^#^	230.82 (69.45)^#^

Data are expressed as mean ± SD or percentage (%). # test *n* = 157, reference *n* = 156

### Primary efficacy endpoint

**Fig. 2:**
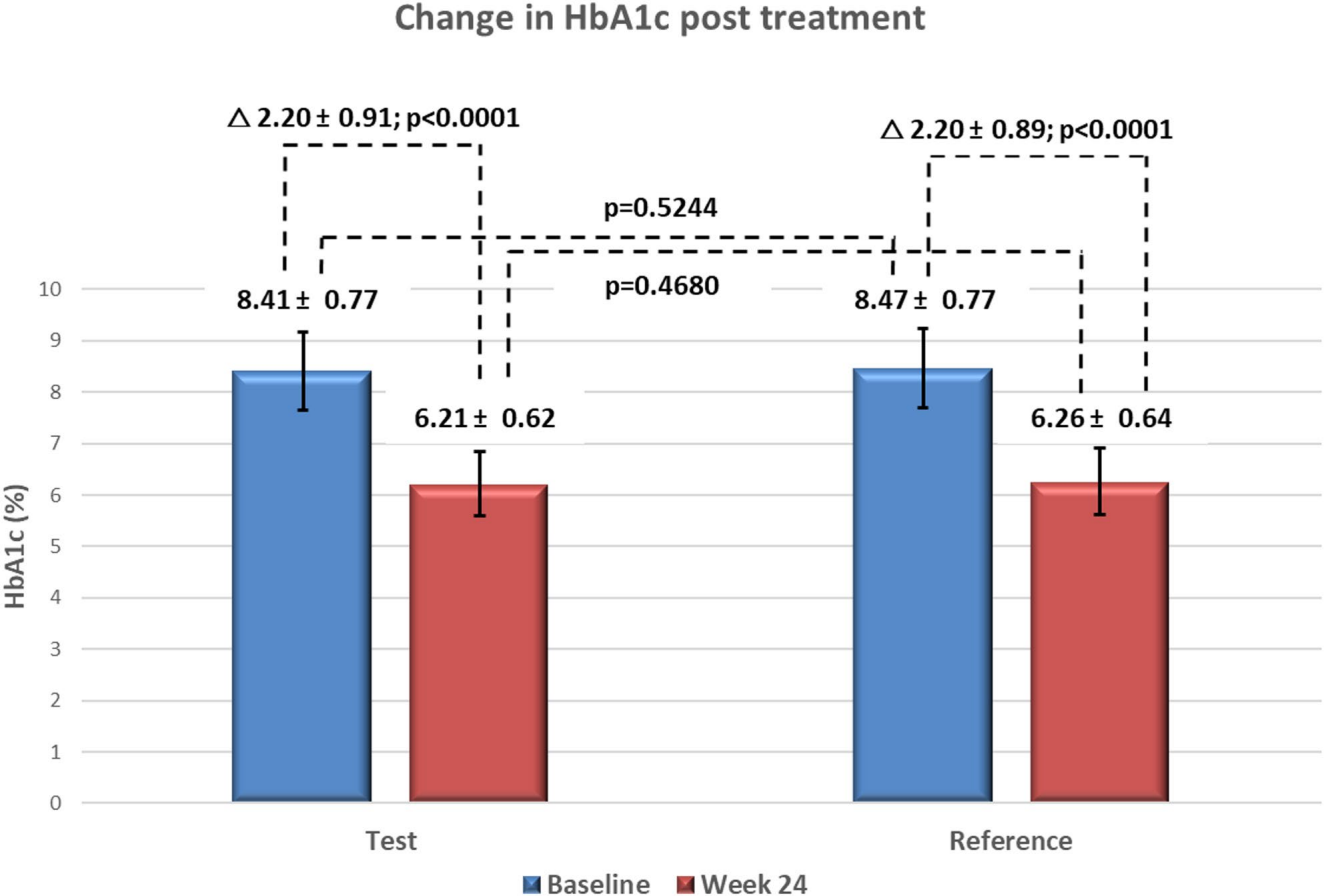
Change in HbA1c (%) before (baseline) and after treatment (week 24) in test and reference arms. Values are mean ± SD; HbA1c, glycated hemoglobin. Within-group reductions were significant (*p* < 0.0001), with no between-group difference

In the per-protocol population, baseline HbA1c levels were similar between the test (8.41 ± 0.77%) and reference (8.47 ± 0.77%) arms (*p* = 0.5244). At Week 24, mean HbA1c levels decreased to 6.21 ± 0.62% in the test arm and 6.26 ± 0.64% in the reference arm, with a mean reduction of -2.20 ± 0.91% and − 2.20 ± 0.89%, respectively (*p* < 0.0001 for both). The between-group difference was not statistically significant (*p* = 0.5089) (Fig. 2).

Least Square Mean (LSM) change in HbA1c was − 2.22% in the test arm and − 2.17% in the reference arm. The LSM difference was − 0.05% (95% CI: -0.19 to 0.09), confirming non-inferiority of the test arm as the upper bound of the CI was well below the pre-specified margin of 0.4%.

### Secondary efficacy endpoints

#### Glycemic parameters

**Fig. 3:**
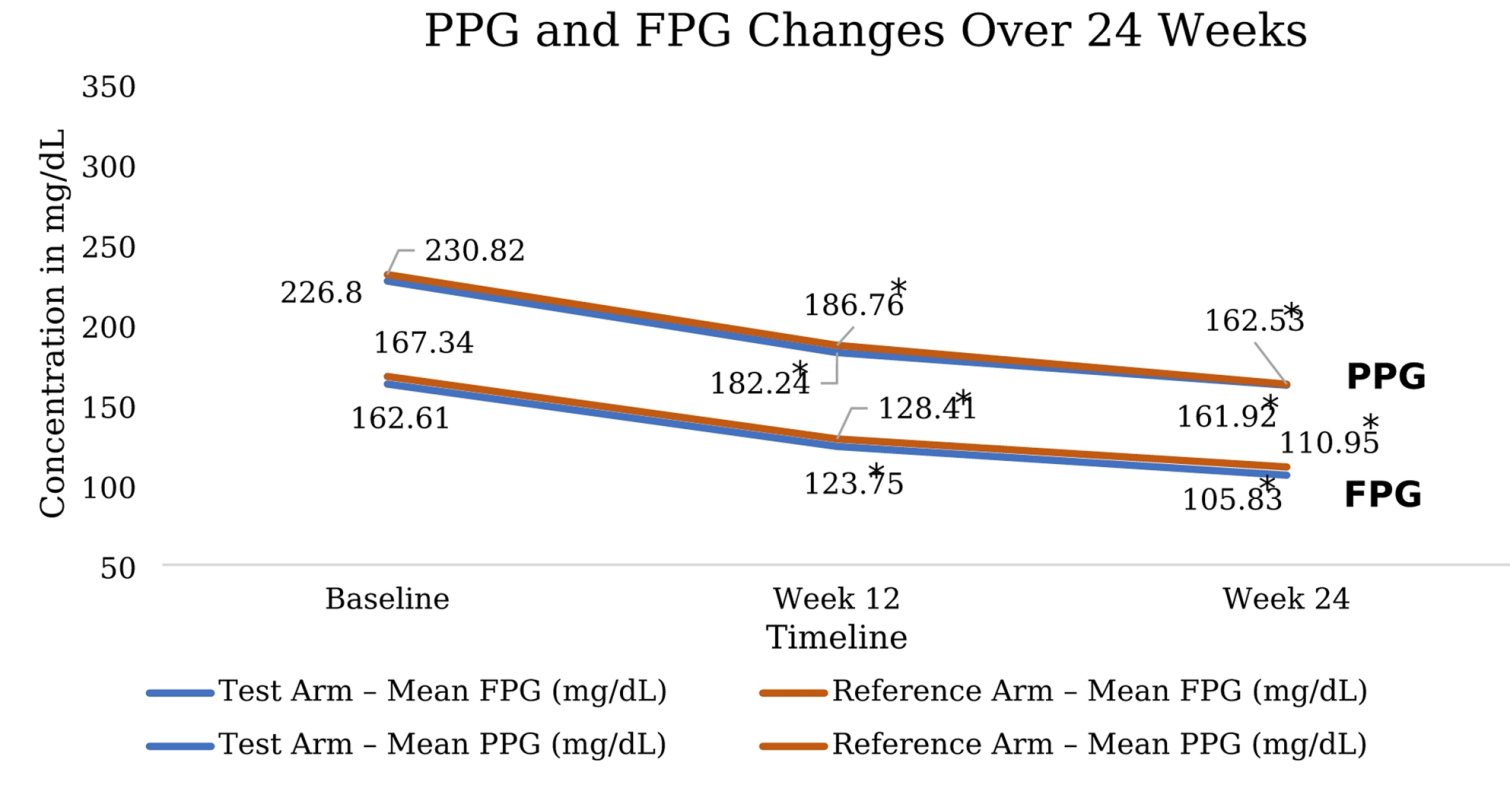
Change in mean fasting plasma glucose (FPG) and post-prandial plasma glucose (PPG) from baseline to Week 24 in the test and reference arms. Values are mean ± SD. Both groups showed significant PPG and FPG reduction from baseline to Week 12 and Week 24 (* *p* < 0.0001), with no significant between-group difference

Baseline fasting plasma glucose (FPG) and post-prandial plasma glucose (PPG) levels were comparable between the test and reference groups. Both treatments produced significant and progressive reductions in FPG and PPG at Week 12 and Week 24 (*p* < 0.0001 for all within-group comparisons). The magnitude of glucose reduction was similar between groups at each time point, with no statistically significant between-group differences. These findings demonstrate comparable glycemic efficacy of the test and reference formulations over 24 weeks. (Fig. 3)

#### Body weight

**Fig. 4:**
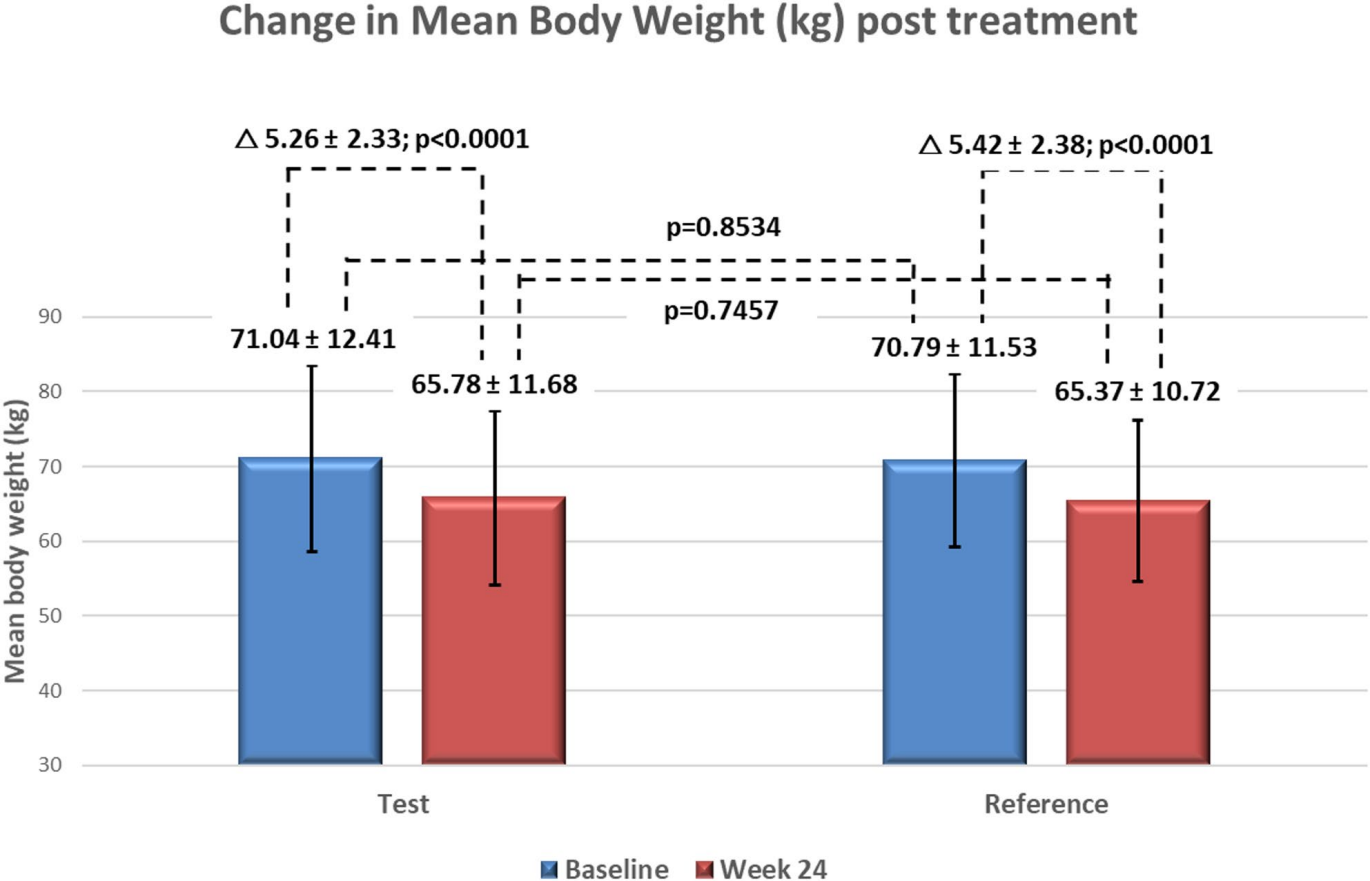
Change in mean body weight (kg) before (baseline) and after treatment (week 24) in test and reference arms. Values are mean ± SD. Both groups showed significant weight reduction from baseline to Week 24 (*p* < 0.0001), with no significant between-group difference

Baseline body weight was 71.04 ± 12.41 kg (test) and 70.79 ± 11.53 kg (reference) (*p* = 0.8534). At Week 24, weight reductions were − 5.26 ± 2.33 kg (test) and − 5.42 ± 2.38 kg (reference) (*p* < 0.0001 for both). The LSM difference was 0.18 kg (95% CI: -0.30 to 0.66; *p* = 0.4588), indicating comparable efficacy (Fig. 4).

#### Proportion achieving HbA1c < 7.0%

The proportion of patients achieving HbA1c < 7.0% increased over time in both arms. At Week 12, 52.23% of patients in the test arm and 57.69% in the reference arm reached this target (*p* = 0.3314). By Week 24, 86.62% and 84.62% of patients in the test and reference groups, respectively, achieved HbA1c < 7.0% (*p* = 0.6125). These proportions should be interpreted in the context of the relatively high baseline HbA1c and the structured dose‑escalation algorithm.

Dose titration followed the predefined scheme in both groups, with most patients up‑titrated beyond 0.5 mg. By Week 24, the distribution of maintenance doses (0.5 mg, 1.0 mg, 2.0 mg) was broadly similar between arms, although detailed dose‑level data were not powered for formal between‑group comparisons. Within each final dose category, descriptive analyses did not reveal systematic differences in HbA1c or weight change between formulations.

### Safety outcomes

A total of 168 treatment-emergent adverse events (TEAEs) were reported in 95 subjects (59.38%) in the test arm, and 165 TEAEs in 81 subjects (50.63%) in the reference arm. Most TEAEs were mild (Grade I): 156 events in 90 subjects (test) and 149 events in 76 subjects (reference). Moderate TEAEs (Grade II) occurred in 10 subjects in each arm.

Three TEAEs remained unresolved at study end (2 in test, 1 in reference). Drug-related TEAEs were reported in 81 subjects (50.63%) in the test arm and 72 subjects (45.0%) in the reference arm. Two serious adverse events (SAEs) occurred in the reference arm; neither was considered related to treatment (Table 2).

**Table 2:** Incidence of treatment emergent adverse events by system organ class (SOC) and preferred term (PT) by treatment group- safety population (*N* = 320)

System Organ Class	Preferred Term	Test (*N* = 160)	Reference (*N* = 160)	Overall (*N* = 320)
Blood and lymphatic system disorders	n (%) E	1 (0.63%)1	0 (0.00%) 0	1 (0.31%)1
	Anaemia	1 (0.63%)1	0 (0.00%) 0	1 (0.31%)1
Ear and labyrinth disorders	n (%) E	1 (0.63%)1	0 (0.00%) 0	1 (0.31%)1
	Ear pain	1 (0.63%)1	0 (0.00%) 0	1 (0.31%)1
Gastrointestinal disorders	n (%) E	74 (46.25%)118	65 (40.63%)118	139 (43.44%)236
	Abdominal distension	1 (0.63%)1	5 (3.13%)5	6 (1.88%)6
	Abdominal pain	3 (1.88%)3	4 (2.50%)4	7 (2.19%)7
	Abdominal pain upper	2 (1.25%)2	1 (0.63%)1	3 (0.94%)3
	Aphthous ulcer	0 (0.00%) 0	1 (0.63%)1	1 (0.31%)1
	Constipation	3 (1.88%)3	8 (5.00%)12	11 (3.44%)15
	Diarrhoea	18 (11.25%)20	15 (9.38%)18	33 (10.31%)38
	Dyspepsia	2 (1.25%)2	1 (0.63%)2	3 (0.94%)4
	Epigastric discomfort	0 (0.00%) 0	1 (0.63%)1	1 (0.31%)1
	Flatulence	3 (1.88%)3	0 (0.00%) 0	3 (0.94%)3
	Gastritis	25 (15.63%)28	19 (11.88%)25	44 (13.75%)53
	Gastrooesophageal reflux disease	3 (1.88%)3	4 (2.50%)5	7 (2.19%)8
	Hyperchlorhydria	7 (4.38%)7	9 (5.63%)10	16 (5.00%)17
	Nausea	21 (13.13%)22	11 (6.88%)12	32 (10.00%)34
	Stomatitis	1 (0.63%)1	0 (0.00%) 0	1 (0.31%)1
	Vomiting	18 (11.25%)23	17 (10.63%)22	35 (10.94%)45
General disorders and administration site conditions	n (%) E	11 (6.88%)12	14 (8.75%)17	25 (7.81%)29
	Asthenia	2 (1.25%)2	3 (1.88%)4	5 (1.56%)6
	Fatigue	1 (0.63%)1	0 (0.00%) 0	1 (0.31%)1
	Pain	0 (0.00%) 0	2 (1.25%)2	2 (0.63%)2
	Pyrexia	8 (5.00%)9	10 (6.25%)11	18 (5.63%)20
Infections and infestations	n (%) E	4 (2.50%)4	5 (3.13%)5	9 (2.81%)9
	Amoebic dysentery	2 (1.25%)2	0 (0.00%) 0	2 (0.63%)2
	Fungal infection	0 (0.00%) 0	1 (0.63%)1	1 (0.31%)1
	Gastroenteritis	0 (0.00%) 0	1 (0.63%)1	1 (0.31%)1
	Influenza	0 (0.00%) 0	1 (0.63%)1	1 (0.31%)1
	Nasopharyngitis	1 (0.63%)1	2 (1.25%)2	3 (0.94%)3
	Rhinitis	1 (0.63%)1	0 (0.00%) 0	1 (0.31%)1
Metabolism and nutrition disorders	n (%) E	7 (4.38%)8	10 (6.25%)10	17 (5.31%)18
	Decreased appetite	7 (4.38%)8	9 (5.63%)9	16 (5.00%)17
	Hypoglycaemia	0 (0.00%) 0	1 (0.63%)1	1 (0.31%)1
Musculoskeletal and connective tissue disorders	n (%) E	4 (2.50%)5	2 (1.25%)2	6 (1.88%)7
	Back pain	0 (0.00%) 0	1 (0.63%)1	1 (0.31%)1
	Myalgia	1 (0.63%)1	0 (0.00%) 0	1 (0.31%)1
	Pain in extremity	3 (1.88%)4	1 (0.63%)1	4 (1.25%)5
Nervous system disorders	n (%) E	11 (6.88%)11	7 (4.38%)7	18 (5.63%)18
	Cerebrovascular accident	0 (0.00%) 0	1 (0.63%)1	1 (0.31%)1
	Headache	7 (4.38%)7	6 (3.75%)6	13 (4.06%)13
	Paraesthesia	4 (2.50%)4	0 (0.00%) 0	4 (1.25%)4
Psychiatric disorders	n (%) E	0 (0.00%) 0	1 (0.63%)1	1 (0.31%)1
	Insomnia	0 (0.00%) 0	1 (0.63%)1	1 (0.31%)1
Respiratory, thoracic and mediastinal disorders	n (%) E	5 (3.13%)5	0 (0.00%) 0	5 (1.56%)5
	Cough	4 (2.50%)4	0 (0.00%) 0	4 (1.25%)4
	Oropharyngeal pain	1 (0.63%)1	0 (0.00%) 0	1 (0.31%)1
Skin and subcutaneous tissue disorders	n (%) E	2 (1.25%)2	4 (2.50%)4	6 (1.88%)6
	Eczema	0 (0.00%) 0	1 (0.63%)1	1 (0.31%)1
	Pruritus	1 (0.63%)1	0 (0.00%) 0	1 (0.31%)1
	Rash	1 (0.63%)1	2 (1.25%)2	3 (0.94%)3
	Skin irritation	0 (0.00%) 0	1 (0.63%)1	1 (0.31%)1
Vascular disorders	n (%) E	1 (0.63%)1	1 (0.63%)1	2 (0.63%)2
	Hypertension	1 (0.63%)1	1 (0.63%)1	2 (0.63%)2

Notes:-[1] Percentages were calculated using respective column header count as denominator. [2] AE= Adverse event; TEAE= Treatment emergent adverse events Medical Dictionary for Regulatory Activities (MedDRA) version 27.0 E= Number of events; N= Number of subjects dosed with each treatment; n= Number of subjects with adverse event with particular Category; %= Calculated using the number of subjects treated with each treatment as the denominator (n/N*100)

The most frequently affected system organ class (SOC) was gastrointestinal disorders, with 118 events reported in both arms. Common GI-related TEAEs (≥ 10%) included diarrhea (10.31%), gastritis (13.75%), nausea (10%), and vomiting (10.94%), consistent with known semaglutide profiles. Incidence rates were comparable between groups.

Laboratory parameters, including renal and hepatic function, pancreatic enzymes, and calcitonin, remained generally stable over 24 weeks, with no clinically meaningful between‑group differences and no laboratory pattern suggesting pancreatitis, drug‑induced liver injury, or thyroid C‑cell pathology.

### Immunogenicity

ADA testing revealed low immunogenicity. At baseline, 1 subject (0.63%) in the test arm and 4 subjects (2.5%) in the reference arm were ADA-positive. Post-treatment, no subjects in the test arm and 2 subjects (1.25%) in the reference arm were ADA-positive. No ADA-positive cases were associated with adverse events or loss of efficacy.

## Discussion

This Phase 3 randomized trial demonstrated that generic semaglutide formulation is non-inferior to the innovator semaglutide, in achieving glycemic control in Indian adults with T2DM inadequately controlled on metformin. The mean HbA1c reduction of -2.20% in both arms over 24 weeks is consistent with prior global studies, including SUSTAIN 1 and STEP 2, which reported reductions ranging from − 1.5% to -2.4% depending on baseline glycemic status and background therapy [7, 8].

The proportion of patients achieving HbA1c < 7.0% at Week 24 (86.62% in test vs. 84.62% in reference) is clinically meaningful and aligns with American Diabetes Association targets for glycemic control [9]. These results are comparable to those observed in real-world studies of semaglutide, which have shown high rates of target attainment even in diverse populations [10].

Secondary endpoints, including reductions in FPG, PPG, and body weight, were statistically significant and similar across both arms. The observed weight loss (~ 5.3 kg) is consistent with findings from STEP 1 and SELECT trials, which demonstrated sustained weight reduction over − 4 years [5, 11]. Weight loss is a critical component of T2DM management, contributing to improved insulin sensitivity and reduced cardiovascular risk [12].

The safety profile of generic semaglutide was favorable, with most adverse events being mild to moderate and consistent with known GLP-1RA class effects. Gastrointestinal events were the most common, including nausea, vomiting, and diarrhea, which are well-documented in semaglutide trials [13]. Importantly, no treatment-related serious adverse events were reported, and the incidence of hypoglycemia was low, supporting semaglutide’s safety when used with metformin [14].

Immunogenicity was minimal, with only a few subjects testing ADA-positive and no clinical consequences observed. This aligns with previous studies showing low immunogenic potential of semaglutide [15].

Affordability remains a major barrier to GLP-1RA use in South-Asian countries. Generic semaglutide may improve access and adherence, especially in public health settings [16]. Real-world data from low- and middle-income countries underscore the importance of cost-effective alternatives to branded therapies [17].

Our study has notable strengths and certain limitations. It is, to our knowledge, the first Phase 3, randomized, active‑controlled, non‑inferiority trial directly comparing a locally manufactured generic semaglutide with the innovator product in Indian adults with type 2 diabetes inadequately controlled on metformin. Conducted across multiple tertiary care centres in diverse geographic regions, the trial enhances the external validity of the findings and reflects routine clinical practice in a real‑world–relevant population. The rigorous design with central randomization, pre‑specified non‑inferiority margin, and concordant results across mITT and per‑protocol populations, supports the robustness of the efficacy and safety conclusions. Demonstrated non‑inferiority in glycaemic control, along with comparable safety and tolerability profiles, highlights the potential of the generic formulation to expand access to GLP‑1RA therapy in resource‑constrained settings where affordability is a key barrier. At the same time, the study has limitations. The 24‑week duration, while sufficient to assess short‑term glycaemic efficacy and initial weight change, does not allow evaluation of the long‑term durability of HbA1c reduction, weight loss, or broader cardiovascular and renal outcomes. The sample size, although adequately powered for the predefined non‑inferiority margin in HbA1c, is not designed to detect rare adverse events or late‑emerging safety signals such as pancreatitis, thyroid C‑cell pathology, or major adverse cardiovascular events. Furthermore, the study population was restricted to adults on metformin monotherapy with defined inclusion and exclusion criteria, which may limit extrapolation to patients with more advanced disease, multiple comorbidities, or complex background therapies. Therefore, larger and longer‑term studies, including cardiovascular and renal outcome trials and real‑world evidence, are needed to confirm the durability of benefits, fully characterize the safety profile of the generic formulation, and further substantiate its role in improving equitable access to GLP‑1RA therapy.

## Conclusion

This Phase 3 trial demonstrated that once-weekly semaglutide achieved clinically meaningful reductions in HbA1c and body weight among Indian adults with type 2 diabetes inadequately controlled on metformin monotherapy. The test formulation proved non-inferior to the innovator product in terms of efficacy and exhibited a comparable safety and tolerability profile, with most patients attaining guideline-recommended HbA1c targets. These findings affirm semaglutide as a viable, cost-effective option for optimizing glycemic management in resource-limited settings. Long-term, real-world studies remain essential to evaluate the durability of these benefits alongside cardiovascular and renal outcomes.

## Supplementary Information

Below is the link to the electronic supplementary material.

Supplementary Material 1

Supplementary Material 2

## Data Availability

The datasets generated and/or analyzed during the current study are available from the corresponding author on reasonable request.

## Abbreviations

BMI: Body mass index
DKA: Diabetic ketoacidosis
ECG: Electrocardiogram
eGFR: Estimated glomerular filtration rate
EREN: Nutritional Epidemiology Research Team
FPG: Fasting plasma glucose
GI: Gastrointestinal
GLP-1RA: Glucagon-like peptide-1 receptor agonist
HbA1c: Glycated hemoglobin
ICH-GCP: International Council for Harmonisation – Good Clinical Practice
MACE: Major adverse cardiovascular events
MEN2: Multiple endocrine neoplasia type 2
mITT: modified intent-to-treat
NT-proBNP: N-terminal pro B-type natriuretic peptide
PP: Per-protocol
PPG: Post-prandial plasma glucose
SAE: Serious adverse event
SD: Standard deviation
SOC: System organ class
TEAE: Treatment-emergent adverse event
T2DM: Type 2 diabetes mellitus
