# Supplementary material for: Efficacy and safety of semaglutide injection in Indian patients with type 2 diabetes mellitus inadequately controlled on metformin: a phase 3, randomized, active-controlled trial (SIZE-DM study)
Source: Cardiovasc Diabetol Endocrinol Rep. 2026 Apr 24;12:21. doi: 10.1186/s40842-026-00290-8 (PMC13107781; doi:10.1186/s40842-026-00290-8)
Supplement: Supplementary file 1 — Supplementary Material 1 [file 40842_2026_290_MOESM1_ESM.docx]

Supplementary file

**Laboratory shift analyses for renal, hepatic, pancreatic enzymes and calcitonin**

The protocol mandated serial assessment of laboratory parameters including renal function (serum creatinine, eGFR), hepatic enzymes (ALT, AST, ALP, total bilirubin), pancreatic enzymes (amylase, lipase), and calcitonin at predefined visits. Shift analyses were performed by categorizing patients according to changes from baseline (e.g., normal to high, high to normal, normal to low) for each analyte over the 24‑week treatment period. Across both treatment arms, the majority of patients remained within reference ranges or exhibited only transient, non‑progressive deviations that did not meet protocol criteria for clinically significant abnormalities or AE reporting. No patient developed serum amylase or lipase ≥3× ULN with clinical features suggestive of pancreatitis, and no clinically meaningful signal for drug‑induced liver injury (ALT/AST ≥3× ULN with elevated bilirubin) was observed. Calcitonin values remained below the exclusion threshold of 50 ng/L for all patients throughout the study, and there were no laboratory patterns suggestive of thyroid C‑cell pathology or progressive renal impairment

**Hematology**

|  | **Screening** | | **Week 24** | |
| --- | --- | --- | --- | --- |
| Parameter | **Test** | **Reference** | **Test** | **Reference** |
| Hemoglobin (gm/dl) | 13.51± 1.97 | 13.55± 1.90 | 13.22± 1.73 | 13.28± 1.88 |
| RBC count (million/mm3) | 4.93± 0.55 | 4.87± 0.68 | 4.83± 0.62 | 4.79± 0.65 |
| Platelet count (/mm3) | 274387.5± 79656.18 | 268120.0± 73416.43 | 289271.6± 72780.74 | 287984.7± 78770.48 |
| Total WBC count (/mm3) | 7796.73± 1803.64 | 7967.97± 2000.44 | 7780.63± 1968.20 | 7937.14± 2135.82 |
| Neutrophil Count (/mm3) | 4609.60± 1256.67 | 4959.86± 1557.16 | 4755.46± 1478.43 | 4859.62± 1723.19 |

**Biochemistry**

|  | **Screening** | | **Week 24** | |
| --- | --- | --- | --- | --- |
| Parameter | **Test** | **Reference** | **Test** | **Reference** |
| Blood Urea Nitrogen (mg/dl) | 11.43± 4.20 | 11.55± 4.25 | 10.71± 3.29 | 10.52± 3.53 |
| eGFR (ml/min) | 102.85± 18.07 | 97.48± 17.0 | 100.96± 16.13 | 101.97± 13.96 |
| Total Bilirubin (mg/dL) | 0.55± 0.26 | 0.57± 0.30 | 0.59± 0.26 | 0.59± 0.35 |
| AST (U/L) | 26.20± 22.42 | 22.08± 8.98 | 23.82± 12.88 | 22.24± 9.35 |
| ALT (U/L) | 26.81± 19.39 | 23.28± 14.44 | 23.95± 17.77 | 21.81± 10.26 |
| ALP (U/L) | 86.97± 32.13 | 91.33± 29.98 | 93.33± 54.10 | 89.33± 35.43 |
| **Creatinine (mg/dl)** | 0.79± 0.18 | 0.84± 0.19 | 0.81± 0.17 | 0.78± 0.16 |
| Sodium (mmol/L) | 138.67± 3.70 | 138.57± 3.25 | 138.14± 3.55 | 138.44± 3.24 |
| Chloride (mmol/L) | 101.02± 4.45 | 100.91± 3.18 | 101.15±3.10 | 101.27± 3.15 |
| Potassium (mmol/L) | 4.37± 0.59 | 4.35± 0.52 | 4.36± 0.46 | 4.42± 0.40 |
| Serum Amylase (U/L) | 68.49± 31.73 | 63.24± 24.81 | 81.18± 142.44 | 69.53± 64.37 |
| Serum Lipase (U/L) | 59.14± 56.27 | 52.41± 46.05 | 65.01± 47.87 | 59.74± 42.66 |
| Serum Calcitonin (ng/L) | 4.58± 4.79 | 4.82± 5.24 | 4.72± 4.78 | 4.36± 3.82 |
