# Supplementary material for: Efficacy and safety of semaglutide injection in Indian patients with type 2 diabetes mellitus inadequately controlled on metformin: a phase 3, randomized, active-controlled trial (SIZE-DM study)
Source: Cardiovasc Diabetol Endocrinol Rep. 2026 Apr 24;12:21. doi: 10.1186/s40842-026-00290-8 (PMC13107781; doi:10.1186/s40842-026-00290-8)
Supplement: Supplementary file 2 — Supplementary Material 2 [file 40842_2026_290_MOESM2_ESM.docx]

|  | **Test** | | | | **Reference** | | | |
| --- | --- | --- | --- | --- | --- | --- | --- | --- |
|  | **0.25 mg** | **0.5 mg** | **1 mg** | **2 mg** | **0.25 mg** | **0.5 mg** | **1 mg** | **2 mg** |
| Visit 2 (Week 0) | 160 | 0 | 0 | 0 | 160 | 0 | 0 | 0 |
| Visit 3 (Week 4) | 0 | 160 | 0 | 0 | 0 | 160 | 0 | 0 |
| Visit 4 (Week 8) | 0 | 77 | 81 | 0 | 0 | 74 | 86 | 0 |
| Visit 5 (Week 12) | 0 | 71 | 48 | 39 | 0 | 69 | 53 | 38 |
| Visit 6 (Week 16) | 0 | 69 | 43 | 45 | 0 | 68 | 49 | 43 |
| Visit 7 (Week 20) | 0 | 69 | 43 | 45 | 0 | 68 | 48 | 41 |
| Visit 8 (Week 24) | 0 | 69 | 43 | 45 | 0 | 67 | 48 | 41 |

**Supplementary table 1: Visit wise patient distribution in test and reference groups as per dosing schedule**
